# Pediatric Balint's Syndrome Variant: A Possible Diagnosis in Children

**DOI:** 10.1155/2016/3806056

**Published:** 2016-11-08

**Authors:** Swetha Sara Philip, Sunithi Elizabeth Mani, Gordon N. Dutton

**Affiliations:** ^1^Department of Ophthalmology, The Cerebral Visual Impairment Clinic, Christian Medical College and Hospital, Vellore, Tamil Nadu 632001, India; ^2^Department of Radiodiagnosis, Christian Medical College, Vellore, Tamil Nadu, India; ^3^Department of Vision Sciences, Glasgow Caledonian University, Cowcaddens Road, Glasgow G4 0BA, UK

## Abstract

Balint's syndrome is well described in adults, but not in children. It is caused by bilateral posterior parietal lobe damage and comprises a triad of simultanagnosia (inability to simultaneously see more than a small number of items), optic ataxia (impaired visual guidance of movement of the limbs and body), and apraxia of gaze (inability to volitionally direct gaze despite the requisite motor substrate) often associated with homonymous lower visual field loss. We, here, describe five children (four males, one female; mean age 7.4 years, [range 4−11 years]; birth weight ≤ 2.5 kg; four were born ≤ 36 weeks of gestational age and one at 40 weeks) who presented to the Cerebral Visual Impairment Clinic at a tertiary care center in South India with clinical features remarkably consistent with the above description. In all children neuroimaging showed bilateral parietooccipital gliosis with regional white matter volume loss and focal callosal thinning, consistent with perinatal hypoxic ischemic encephalopathy and possible neonatal hypoglycemia.

## 1. Introduction

Balint's syndrome is well described in adults [1]. It is characterized by a triad of visual spatial difficulties related to simultanagnosia (inability to simultaneously see more than a small number of items at any one time) [2], optic ataxia (impaired visual guidance of movement including reach) [3], and psychic paralysis of gaze (inability to volitionally direct visual gaze despite the requisite motor substrate) [1, 4]. Balint's syndrome is commonly accompanied by homonymous lower visual field loss. Neuroimaging in Balint's syndrome shows bilateral posterior parietal lobe damage due to a range of causes [5]. Balint's syndrome, in children, is rarely recognized and reported, and to our knowledge there are two reported cases in literature [3, 6]. We have identified 5 children who presented to the Cerebral Visual Impairment (CVI) clinic in a tertiary care center in South India between 2011 and 2012 with visual behaviors consistent with that of Balint's syndrome.

This retrospective observational study was done after obtaining permission from the institutional review board. These children were referred to the clinic by their clinician, who provided salient information regarding their neurological/cognitive status. A pediatric ophthalmologist trained in CVI vision assessment assessed these children. The clinical assessment began the moment children walked into the waiting room with their parents/caregivers. Spontaneous visual responses like what the child was looking at, the size, the nature of the object, and the distance at which the child appreciated the object of regard were closely observed. Visual-perceptual disorders were initially identified, based on in-depth history taking, using spontaneously volunteered information about visual behaviors, and also using the Structured Clinical Question Inventory [7]. Detailed history taking revealed that all five children had symptoms of visual-cognitive-perceptual dysfunction. The radiologist provided radiological interpretations.

**Figure 1:**
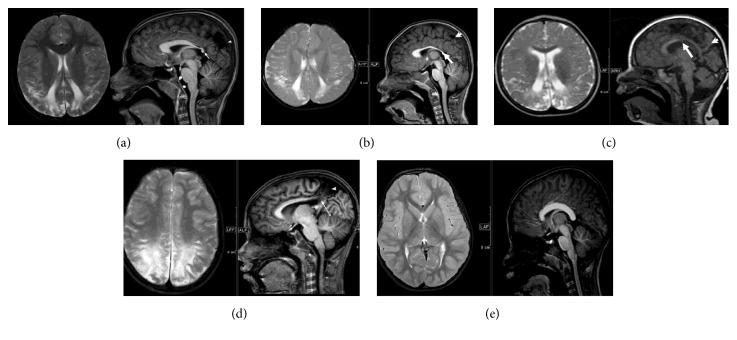
T2 axial and T1 sagittal MRI brain scans of the 5 children described. Cases (a)–(c)—bilateral symmetric parietooccipital gliosis (arrowhead) with regional white matter volume loss, thinning in the posterior body, isthmus, and splenium of the corpus callosum (white arrow) being evident. Associated white matter hyperintensities and ventricular dilatation are seen. Case (d)—bilateral symmetric parietal gliosis was seen (arrowhead) with marked thinning of the isthmus and splenium of the corpus callosum (long white arrow). Case (e)—a near normal scan with prominence of the atria and mild shortening of the corpus callosum and splenium, with thinning of the splenium.

The parents/caregivers described their children as being “clumsy and careless” in their activities of daily living. All had difficulty in reading and copying the text. They were “poor performers” in school with three of them dropping out of school as they could not “cope” with the school environment. All five would probe the floor before they took a confident step as they had difficulty negotiating steps, stairs, pavements, and unfamiliar patterned floors. All five children had features suggestive of optic ataxia of upper limb by placing objects inaccurately on the edge of the table [4]. Crowded places caused a great deal of discomfort and distress to all the children and they found finding objects against a patterned background an uphill task. They had difficulty dressing themselves (e.g., often donning clothes inside out) [4, 8]. During the assessment it was observed that all the children had wide-open midflight of their fingers while reaching out for a color pencil, books, or a toy and had a tendency to place objects inaccurately on the edge when replacing toys and books on the table. These features were consistent with optic ataxia of upper limbs. While walking around in the play area they were seen to be colliding with low-lying benches even when looking towards them consistent with impaired visual guidance of the lower limbs. In all cases, each child had difficulty initiating saccades (apraxia of gaze) when asked to seek and reach out for a toy or look towards a sound in spite of ocular motility being full and absence of strabismus. Involvement in a friendly conversation while doing a task at the same time made these children irritated due to their inability to split attention. None could identify nor find the picture book placed among an untidy pile of books and loose papers. They picked up whichever book they found on the top. They were more comfortable when the table was more organized and had less clutter. Two children could identify a “toy” through but could not through vision, indicative of additional ventral stream dysfunction [7]. All five children had difficulties identifying shapes. Neuroimaging (Figures 1(a)–1(e)) showed bilateral posterior parietal and occipital gliosis with regional white matter volume loss and focal callosal thinning in all the children. The profiles of the individual cases are presented below and also in Table 1.

## 2. Case Presentations

### 2.1. Case 1

An 8-year-old boy, born of caesarian section (mother had pregnancy induced hypertension) at 32 weeks of gestation with a birth weight of 1.2 kg, neonatal period being uneventful, was brought to the CVI clinic with complaints of being a poor performer in school. He needed help in performing his activities of daily living (ADL) like dressing himself up and tying his shoes. He had to drop out of school, as he was not able to cope with busy school environment and was termed a “bad boy.” His magnetic resonance imaging (MRI) showed bilateral parietooccipital gliosis with corpus callosal thinning (Figure 1(a)). Ophthalmological examination showed best-corrected visual acuity (BCVA) with Snellen's visual acuity chart being 6/60 (binocularly) with a myopic correction, divergent squint, and no nystagmus and dilated fundus examination was normal. He did not like wearing his spectacles. On CVI assessment it was noted that he had difficulty reading and copying text. He had lower field defect and right-sided neglect. He exhibited features suggestive of simultanagnosia, apraxia of gaze, optic ataxia, and impaired visual guidance of lower limbs.

### 2.2. Case 2

A 4-year-old boy referred to the clinic for being “clumsy.” He had been born at 40-week gestation (pregnancy was uneventful) by spontaneous vaginal delivery and birth weight of 2.5 kg. There is history suggestive of birth asphyxia. MRI showed biparietooccipital gliosis with callosal thinning (Figure 1(b)). He was slow and clumsy in performing his activities of daily living (ADL). He was attending a kindergarten but was being a “nuisance” to his teachers and peers. He preferred quiet environment and would harm his friends if they became too noisy. He did not like watching television. His BCVA with Cardiff cards was 6/48 (binocularly) with no refractive error and ophthalmology examination including slit-lamp biomicroscopy and dilated fundus examination within normal limits. He had features suggestive of simultanagnosia, optic ataxia, apraxia of gaze, and impaired visual guidance of upper and lower limbs and lower field defect.

### 2.3. Case 3

A 7-year-old boy, born by spontaneous vaginal delivery, at 36 weeks of gestation with a birth weight of 1.8 kg and history of respiratory distress at birth, was seen in the CVI clinic with complaints of poor performance in school. He developed seizure disorder and spastic diplegia. He was able to carry out self-care skills to a large extent on his own. He never liked going to school, as he could not keep up with reading and writing work. He spent most of his free time playing with his toys or coloring pictures. He got upset when furniture in the house or his toys were rearranged. He could not appreciate fast moving things and watched only music channels on television. Ophthalmological examination showed BCVA (binocular, Snellen's visual acuity chart) 6/24 with alternate divergent squint; the rest of the anterior and dilated posterior segment examination was unremarkable. On CVI assessment, he had features suggestive of lower field defect, simultanagnosia, optic ataxia, apraxia of gaze, and impaired visual guidance of upper limbs. Radiological features showed biparietal occipital gliosis (Figure 1(c)).

### 2.4. Case 4

An 11-year-old boy, with a BCVA (binocular) of 6/6 with Snellen's visual acuity chart and the rest of the ophthalmological examination being within normal limits, was assessed in the CVI clinic for his learning and intellectual disability. The pregnancy was uneventful and he was born at 32 weeks of gestation by caesarian section. His birth weight was 2.25 kg and had history suggestive of perinatal asphyxia. At 7 years of age he developed seizure disorder. He was mostly independent in self-care skills and was studying in Grade 1. CVI assessment showed that he had difficulty in handling complex visual scene, problems with clutter and impaired visual guidance of hands, body, and legs, and lower field defect. He knew his letters, but when asked to write he was very hesitant and wrote vertically downwards, missing a few letters in between. He had difficulty with recognition of faces or shapes or objects. His MRI is shown in Figure 1(c).

### 2.5. Case 5

A 7-year-old girl was seen in CVI clinic for her intellectual disability and poor performance in school. She was born from an uneventful pregnancy at 36 weeks of gestation by spontaneous vaginal delivery with a birth weight of 2.0 kg. Her neonatal period was uneventful. She was able to carry out her activities of daily living on her own, but owing to her “careless” nature her parents did most of the work for her. She had to discontinue her school education, as she did not cope with school environment. She lacked confidence and needed constant prompting while carrying out any tasks. While walking on the pedestrian path she always tugged at her parents clothes. She would bump into objects like trees or electric pole while having a conversation with her parents when out on a walk. Her social interaction was poor as she disliked crowded places and avoided all social gatherings. She watched television from very close distance and watched only slow moving cartoon shows. Ophthalmology examination showed BCVA (binocularly) with Snellen's visual acuity chart being 6/24 with the rest of the examination including dilated fundus examination being within normal limits. On CVI assessment, she had features suggestive of lower field defect, simultanagnosia, optic ataxia, apraxia of gaze, and impaired visual guidance of upper limbs. Her MRI scans showed a near normal scan with only mild shortening of the corpus callosum and splenium and thinning of the splenium (Figure 1(d)).

## 3. Discussion

Cerebral Visual Impairment (CVI) comprises visual malfunction due to retrochiasmatic primary visual and visual association pathway pathology. This can be isolated or accompany anterior visual pathway dysfunction [7]. It is a major cause of low vision in children in the developed and developing world due to increasing survival in pediatric and neonatal care [8]. Most children with CVI have other neurological problems, but when it occurs in isolation the diagnosis can be overlooked and go unrecognized. There are two principal higher visual pathways involved in CVI, the dorsal stream and the ventral stream [9]. The dorsal stream passes between the occipital lobes and the posterior parietal lobes. The posterior parietal lobes subconsciously subserve the function of handling complex three-dimensional visual scenes. These, along with the frontal and temporal lobes, are involved in planning movement with accuracy, perceiving locating surrounding items, and bringing about fast eye movements to look at those of interest [7]. The symptoms of dorsal stream dysfunction are varied in character and severity. It is this pathway that is damaged in Balint's syndrome, while associated damage with the superior optic radiations leads to concomitant lower visual field impairment [10, 11]. Additional impairment in recognition despite the requisite visual acuity is likely to be due to damage to the pathway between the occipital and temporal lobes, the ventral stream. The term “dorsal stream dysfunction plus” has recently been suggested to cover dorsal stream dysfunction with additional impaired recognition [9]. Balint's syndrome has a varied etiology including stroke [5], brain injury [1], and infection and progressive multifocal leukoencephalopathy [4]. Balint's syndrome in children is probably not uncommon but has been infrequently reported, perhaps because it is rarely being recognized and diagnosed [3, 6]. Most literature on Balint's syndrome is research based. All three features of simultanagnosia, impaired visual guidance of movement, and psychic paralysis of gaze, accompanied by lower visual field impairment and radiological features, were evident in the 5 children described, constituting the clinical picture of Balint's syndrome [6]. Structured history taking helped to reveal this diagnosis.

Habilitation approaches suggested to these patients involved simple multicontext adaptive strategies that made use of the strengths of the patients to compensate for their difficulties as described in the literature [12]. It is important to help the patients and their caregivers to understand their child's functional difficulties and the strategies suggested, so as to enhance their child's abilities to overcome the difficulties. This understanding also helps parents and care givers to support the child and avert criticism of their poor performance in visual search and their apparent clumsiness. In conclusion, this poorly recognized condition in children with visual-cognitive-perceptual difficulties, though common in our experience, needs to be identified so as to better understand and manage the condition through simple targeted strategies.

## Figures and Tables

**Table 1:** Profiles of children with features of Balint's syndrome.

	Case 1	Case 2	Case 3	Case 4	Case 5
Age at presentation to the clinic	8 years	4 years	7 years	11 years	7 years
Gender	Male	Male	Male	Male	Female
Gestational age	32 weeks	40 weeks	36 weeks	32 weeks	36 weeks
Birth weight	1.2 kg	2.5 kg	1.8 kg	2.25 kg	2.0 kg
Antenatal complications	Pregnancy induced hypertension (PIH)	Nil	Nil	Nil	Nil
Effect of visual disability	Poor performance at school	Clumsiness	Poor performance at school	Poor performance at school	Poor performance at school
Visual acuity	6/60	6/48	6/24	6/6	6/24
Fundus	Normal	Normal	Normal	Normal	Normal
Simultanagnostic visual dysfunction	Present	Present	Present	Present	Present
Optic ataxia	Present	Present	Present	Present	Present
Apraxia of gaze	Present	Present	Present	Present	Present
Problems with clutter, crowd, and lower field defect	Present	Present	Present	Present	Present
Difficulties recognizing faces, words, shapes, and objects	Present	Present	Present	Present	Present
Radiological findings	Biparietooccipital gliosis + corpus callosum thinning	Biparietooccipital gliosis + corpus callosum thinning	Bilateral occipital gliosis + corpus callosum thinning	Biparietal gliosis + corpus callosum thinning	Mild posterior parietooccipital gliosis
